# Unintentional non-adherence to chronic prescription medications: How unintentional is it really?

**DOI:** 10.1186/1472-6963-12-98

**Published:** 2012-06-14

**Authors:** Abhijit S Gadkari, Colleen A McHorney

**Affiliations:** 1 U.S. Outcomes Research, Merck & Co., Inc, 351 North Sumneytown Pike, North Wales, PA, 19454, USA

## Abstract

**Background:**

Unintentional non-adherence has been characterized as passively inconsistent medication-taking behavior (forgetfulness or carelessness). Our objectives were to: (1) study the prevalence and predictors of unintentional non-adherence; and (2) explore the interrelationship between intentional and unintentional non-adherence in relation to patients’ medication beliefs.

**Methods:**

We conducted a cross-sectional survey of adults with asthma, hypertension, diabetes, hyperlipidemia, osteoporosis, or depression from the Harris Interactive Chronic Illness Panel. The analytic sample for this study included 24,017 adults who self-identified themselves as persistent to prescription medications for their index disease. They answered three questions on unintentional non-adherence (forgot, ran out, being careless), 11 questions on intentional non-adherence, and three multi-item scales assessing perceived need for medication (k = 10), perceived medication concerns (k = 6), and perceived medication affordability (k = 4). Logistic regression was used to model predictors of each unintentional non-adherence behavior. Baron and Kenny’s regression approach was used to test the mediational effect of unintentional non-adherence on the relationship between medication beliefs and intentional non-adherence. Bootstrapping was employed to confirm the statistical significance of these results.

**Results:**

For the index disease, 62% forgot to take a medication, 37% had run out of the medication, and 23% were careless about taking the medication. Common multivariate predictors (p < .001) of the three behaviors were: (1) lower perceived need for medications; (2) more medication affordability problems; (3) worse self-rated health; (4) diabetes or osteoporosis (relative to hypertension); and (5) younger age. Unique statistically-significant predictors of the three behaviors were: (a) ‘forgot to take medications’ - greater concerns about the index medication and male gender; (b) ‘run out of medications’ - non-white race, asthma, and higher number of total prescription medications; (c) ‘being careless’ - greater medication concerns. Mediational tests confirmed the hypothesis that the effect of medication beliefs (perceived need, concerns, and affordability) on intentional non-adherence is mediated through unintentional non-adherence.

**Conclusions:**

For our study sample, unintentional non-adherence does not appear to be random and is predicted by medication beliefs, chronic disease, and sociodemographics. The data suggests that the importance of unintentional non-adherence may lie in its potential prognostic significance for future intentional non-adherence. Health care providers may consider routinely inquiring about unintentional non-adherence in order to proactively address patients’ suboptimal medication beliefs before they choose to discontinue therapy all together.

## Background

Medication adherence refers to the extent of conformity to treatment recommendations with respect to the timing, dosage, frequency, and duration of a prescribed medication [1]. Non-adherence to chronic medications is an Achilles’ heel of evidence-based medicine and is one of the major reasons why patients fail to reach their clinical goals, resulting in suboptimal health outcomes [2,3]. In an effort to better understand the underlying causes of patients’ medication-taking behaviors, researchers have differentiated between two types of non-adherence. Intentional non-adherence is an active decision on the part of patients to forego prescribed therapy [4,5]. Unintentional non-adherence, on the other hand, is a passive process whereby patients fail to adhere to prescribing instructions through forgetfulness, carelessness, or circumstances out of their control (e.g., health literacy) [6,7]. Patients can, and often do, exhibit both types of non-adherent behaviors [8-10]. Intentional non-adherence may be demonstrated through non-fulfillment of a new prescription or through discontinuation of an existing medication therapy without the advice of a provider. Approximately 15% of patients do not fill a new prescription [11]; of those who do fill a new prescription, roughly 50% discontinue therapy in the first six months [3,12,13]. Estimates of unintentional non-adherence vary considerably and range from 20% to over 50% [7,8,10,14-23].

Research over the past 20 years has consistently shown that intentional non-adherence is driven by patient beliefs about their treatment, disease, and prognosis as well as their objective experiences with medications [24-27]. Some early research on unintentional non-adherence suggested it was more strongly correlated with demographic characteristics [6,7,28,29] than with medication knowledge or beliefs. Recent work, however, indicates that that there may be more to unintentional non-adherence than pure forgetfulness or carelessness [10,15,20,21,30,31]. Patients reporting intentional versus unintentional non-adherence have been found to be similar to one another in terms of their adherence-related knowledge and motivation [31]. Unintentional non-adherence has also been recently linked to perceived need for medications [10,30], medication concerns [15,19,21], and beliefs about treatment efficacy [20], which suggests that interventions focusing on patients’ medication beliefs may be required to address both intentional and unintentional non-adherence.

Although recent studies have begun to clear the veil on unintentional non-adherence, the generalizability of the evidence to date is limited by small samples in niche populations. A majority of these results were reported in samples of about 150 patients or less [9,10,15,20,30], typically for a single condition [9,10,15,20,31], and usually with a single-item measure of unintentional non-adherence [15,20,30,31]. The one study that was conducted in multiple chronic diseases with somewhat of a larger sample only targeted Medicare enrollees and excluded uninsured patients or those enrolled primarily in private/employer-sponsored health plans [21]. Finally, although it has been documented that patients exhibit both intentional and unintentional non-adherence, no previous study has explored the interrelationship between unintentional and intentional non-adherence in relation to patients’ medication beliefs. The current study, conducted in a large sample of adults with chronic disease, aimed to: (1) report the prevalence of unintentional non-adherence; (2) explore whether medication beliefs predict unintentional non-adherence; and (3) explore the interrelationship between unintentional and intentional non-adherence in relation to medication beliefs.

## Methods

### Study population

A cross-sectional survey of U.S. adults with self-reported chronic disease was conducted using the Harris Interactive Chronic Illness Panel (CIP). CIP participants are recruited through a number of avenues, including postal-mail invitations, television advertisements, telephone recruitment for under-represented populations, targeted email solicitations, and text-banner placements on websites (e.g., social-media, news sites, search-engine sites and community portals). In February and March of 2009, randomly-selected members of the CIP were sent an e-mail invitation to participate in the survey. Panel members responding to the e-mail invitation were instructed to read the informed consent form and click on *yes* if they agreed to participate. The study was approved by the George Mason University Institutional Review Board.

Panel members were eligible for participation if they were aged 40 and older, resided in the U.S., and reported one of six chronic diseases (hypertension, hyperlipidemia, diabetes, asthma, osteoporosis, or depression). Only persons age 40 and older were sampled because they bear a greater burden of chronic disease compared to adults aged 18–39. The six chronic diseases reflect a mix of symptomatic, asymptomatic, and mental-health conditions. They are some of the most highly prevalent conditions in the U.S. [32] and are associated with a significant clinical and economic burden for the U.S. health care system [33].

Requests for survey participation were sent to 204,266 randomly-selected CIP members. Of these invitations, there were 11,945 invalid e-mail addresses. Of the 192,321 invitations with valid e-mail addresses, 17,121 were ineligible due to age less than 40. Of the 175,200 age-eligible persons with a valid e-mail address, 51,774 completed the screener (29.5% survey contact rate per standards recommended by The American Association for Public Opinion Research [34]).

During the screening portion of the survey, panel members’ chronic disease status was reconfirmed and their medication-taking behavior for the index condition was solicited. The screener solicited the number of medications respondents currently took for each disease as well as the length of time they reported continuously taking the medication. These items were used to classify respondents as currently persistent to their medication. To identify respondents as non-persisters, the survey asked if, in the last year, they had stopped taking a prescription medication for one of the six conditions without their providers telling them to do so. To identify respondents as non-fulfillers, the survey asked if, in the last year, they had received, but did not fill, a new prescription from their provider for one of the six target conditions. Only subjects who self-identified themselves as persistent to (i.e., currently on therapy with) prescription medications for their index disease form the analytic sample for this study. The index disease was the disease for which respondents completed the medication-belief items. If self-reported persisters reported more than one of the six target conditions, one was randomly selected as the index disease. Because non-fulfillers never filled the index medication, they were not asked questions about unintentional non-adherence. Self-reported non-persisters were excluded from the analytic sample because their complete discontinuation of therapy cannot, logically, be unintentional.

### Survey content

Study participants completed a core set of questions on demographics and self-reported health status. They answered three questions on unintentional non-adherence in reference to the prescription medications for their index disease: During the past six months: (1) did you ever forget to take the prescription medication; (2) did you ever run out of the prescription medication; and (3) were you careless at times about taking the prescription medication? Respondents also answered 11 questions on intentional non-adherence in reference to their medication-taking behavior in the past six months for their index disease. These questions surveyed the respondents on the following behaviors: took less medication than instructed because they felt better/worse; skipped taking medication because they felt better/worse; altered dose of medication to suit own needs; stopped taking medication because they felt better/worse; skipped doses to make medication last longer; took smaller doses to make medication last longer; skipped doses of medication because they did not think it was helping them; and stopped medication because they did not think it was helping them. Both sets of measures of unintentional and intentional non-adherence were adapted from validated measures published in the peer-review literature [35-39].

Finally, respondents answered 20 questions assessing their beliefs about the index medication: perceived need for the medication (k = 10), perceived medication concerns (k = 6), and perceived medication affordability (k = 4). As described elsewhere [26], multi-item scales were created by summing raw items into a scale score and linearly transforming each sum to a 0–100 metric, with 100 representing the most favorable belief (highest perceived need, fewest perceived concerns, fewest affordability issues), 0 the least favorable, and scores in between representing the percentage of the total possible score. Each multi-item scale was highly reliable [26]. Included in the 20 items were the three items from the Adherence Estimator®, a brief, three-item adherence screener. One item each from the Adherence Estimator® assessed the domains of perceived need for medications, perceived medication concerns, and perceived medication affordability [26].

### Analysis

Logistic regression was used to assess differences between age-eligible CIP members with valid e-mail addresses who did and did not responded to the screener (survey non-contact bias per standards recommended by The American Association for Public Opinion Research [34]). Independent variables were age, gender, race, education, income, geographic region of residence, and chronic disease. Prevalence of unintentional and intentional non-adherence among self-identified persisters was tabulated. Chi-square tests were used to assess for variation of *any* unintentional non-adherence across key variables. Multivariate logistic regression models were used to predict each of the three unintentional non-adherence behaviors (dichotomized as yes/no) as well as *any* unintentional non-adherence behavior using the multi-item scales for perceived need, perceived concerns, and perceived affordability as independent variables. To assess whether the relationship between medication beliefs and unintentional non-adherence was linear and monotonic, we divided each multi-item scale into quartiles and modeled them as dummy variables. We also modeled unintentional non-adherence using the Adherence Estimator® total score (0–36) and risk levels for non-adherence (low, medium, high) based on the total score. As described elsewhere [26], respondents were classified as low risk for non-adherence if they had a total score of 0, medium risk if they had a score of 2–7, and high risk for non-adherence if they had a score of 8 or higher. Covariates in the models included age, gender, race, education, income, index disease, employment status, and self-rated health. All analyses were conducted using Stata SE version 10.

**Figure 1:**
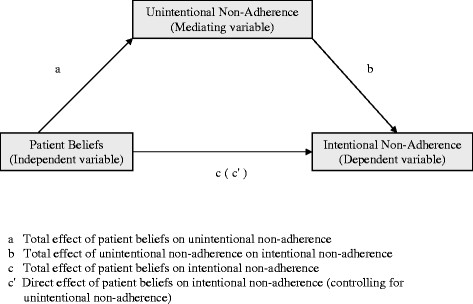
Conceptual Diagram Showing the Mediating Effect of Unintentional Non-Adherence.

We hypothesized that the effect of medication beliefs (perceived need, perceived concerns, and perceived affordability) on intentional non-adherence would be mediated through unintentional non-adherence. In other words, it was hypothesized that medication beliefs would significantly impact unintentional non-adherence which, in turn, would significantly influence their intentional non-adherence (Figure 1).

The most commonly-used method for testing mediation hypotheses is the causal steps approach by Baron and Kenny [40]. For a variable to be considered a mediator according to this approach, four conditions must be met [40,41]: (1) the independent variable must be significantly associated with the mediator—path a in Figure 1; (2) the mediator must be significantly associated with the dependent variable—path b in Figure 1; (3) the independent variable must be significantly associated with the dependent variable—path c in Figure 1; and (4) the direct effect of the independent variable on the dependent variable after controlling for the mediator must be less than the total effect without controlling for the mediator—path c’ in Figure 1 should have a smaller coefficient than c. These four conditions were tested using four linear regression models. For this analysis, two separate multi-item scales were created for unintentional and intentional non-adherence by summing raw items (three items for unintentional non-adherence and 11 items for intentional non-adherence) into a scale score and linearly transforming each sum to a 0–100 metric, with 100 representing the highest level of non-adherence (positive response for all items), 0 representing complete adherence (negative response on all items), and scores in between representing the percentage of the total possible non-adherence score.

The regression approach proposed by Baron and Kenny [40] involves an implicit assumption that the estimates of the indirect effect are normally distributed. There are reasons to suspect that this assumption may not always hold when mediation is present as indirect effects are usually positively skewed and kurtotic [42-44]. A nonparametric resampling procedure called bootstrapping has gained popularity in research on mediation [45-47] because it makes no assumption about the shape of the sampling distribution of the indirect effect. Simulation research has shown that bootstrapping is one of the most valid and powerful methods for testing mediation effects [43,48-52]. With bootstrapping, one draws several thousand samples from the original data (of equal size to the original sample), each of which is obtained by random sampling with replacement from the original sample. As recommended by Hayes and others [41,46], we conducted a bootstrapping procedure with 5,000 samples. These samples were then used to compute the mediated effect and its standard error. Finally, the probability distribution from all of the resampled estimates was used to calculate bias-corrected confidence intervals and bootstrap-estimated standard errors of the mediated effects [43]. If the confidence interval did not include a zero, then a significant indirect effect was concluded [41]. Adjustments for multiple comparisons were made using the Bonferroni correction approach [48].

## Results

### Survey non-contact bias

A 29.5% survey contact rate was achieved. Compared to those age-eligible respondents with valid e-mail addresses who were invited but did not respond to the screener, those successfully contacted were more likely to be male, age 50 and older, Caucasian, and of higher income but were less likely to have asthma or depression (data not shown). Sociodemographic characteristics (age, gender, race, education, and income), geographic region, and chronic diseases explained only 3.2% of the variance (Cox and Snell pseudo R-square test) in panel members’ likelihood of completing the screener.

### Sample description

Of the respondents, 24,017 subjects (88%) self-identified themselves as persistent to the prescription medication for the index disease. Data on these respondents was included in the current analysis. As shown in Table 1, a majority of the self-reported persisters were white (95%), age 60 years and older, and college educated. Two-thirds reported their health as good, very good, or excellent. Over 42% reported hypertension as their index disease, followed by hyperlipidemia (19%), diabetes (13%), osteoporosis (13%), asthma (7%), and depression (5%). Overall, the respondents had a relatively high perceived need for medications (mean of 81.9), moderate medication concerns (mean of 66.7) and moderate perceived medication affordability (mean of 61.4).

**Table 1:** Sample Description (N = 24,017)

**Variable**	**N**	**%**
**Age**, Mean (SD)	61.3 (9.4)	
40–49	2,415	10.1%
50–59	7,899	32.9%
60–64	4,922	20.5%
65–69	4,028	16.8%
70+	4,753	19.8%
**Gender**		
Male	11,631	48.4%
Female	12,386	51.6%
**Education**		
High school or less	4,211	17.5%
Some college	9,314	38.8%
College degree	4,178	17.4%
> College degree	6,314	26.3%
**Income**		
< 25 K	3,143	14.7%
≥ 25K < 50K	5,862	27.4%
≥50K < 75K	4,884	22.9%
≥75K < 100K	3,252	15.2%
≥100K	4,215	19.7%
**Race**		
White	22,421	94.5%
Black	521	2.2%
Hispanic	176	0.7%
Other	620	2.6%
**Health**		
Fair/Poor	7,934	33.0%
Good	10,028	41.7%
Very good/Excellent	6,055	25.2%
**Index Chronic Disease/Medication**		
Asthma	1,773	7.4%
Diabetes	3,098	12.9%
Hyperlipidemia	4,639	19.3%
Hypertension	10,199	42.5%
Osteoporosis	3,073	12.8%
Depression	1,235	5.1%
	**Mean**	**SD**
**Medication Beliefs Scores**^a^		
Perceived need for medications	81.9	14.6
Perceived medication concerns	66.7	21.9
Perceived medication affordability	61.4	30.1

^a^ Multi-item scales were created for medication beliefs scales by summing raw items into a scale score and linearly transforming each sum to a 0–100 metric, with 100 representing the most favorable belief (highest perceived need, fewest perceived concerns, highest perceived affordability), 0 the least favorable, and scores in between representing the percentage of the total possible score.

### Prevalence of unintentional and intentional non-adherence

An overwhelming majority (70%) of the self-reported persisters reported at least one of the three unintentional non-adherence behaviors in the past six months (Table 2). Forgetting to take medication (62%) was the most commonly-reported behavior followed by running out of medications (37%) and being careless at times about taking the medication (23%). Just over one-third of the respondents reported at least one of the eleven intentional non-adherence behaviors in the previous six months. Skipping doses to make medication last longer was the most commonly reported intentional non-adherence behavior (18%), followed by taking smaller doses to make medication last longer (15%), and altering dose of medication to suit own needs (14%).

**Table 2:** Prevalence of Unintentional and Intentional Non-adherence (N = 24,017)

**Unintentional Non-adherence**		
**Items**	**N**	**%**
Ever forget to take medication in past six months	14,987	62.4%
Ever run out of medication in past six months	8,861	36.9%
Careless at times about taking medication in past six months	5,522	23.0%
Reported at least one of the three unintentional non-adherence behaviors in the past six months	16,832	70.1%
**Intentional Non-adherence**		
**Items**	**N**	**%**
Skipped doses to make medication last longer	4,265	17.8%
Took smaller doses to make medication last longer	3,616	15.1%
Altered dose of medication to suit own needs	3,448	14.4%
Stopped taking medication because felt worse	2,071	8.6%
Took less medication because felt better	1,922	8.0%
Skipped medication because felt worse	1,862	7.8%
Skipped medication because felt better	1,766	7.4%
Stopped medication because didn’t think it was helping	1,785	7.4%
Took less medication because felt worse	1,665	6.9%
Stopped taking medication because felt better	1,529	6.4%
Skipped doses because didn’t think medication was helping	1,466	6.1%
Reported at least one of the eleven intentional non-adherence behaviors in the past six months	8,235	34.3%

### Bivariate associations with any unintentional non-adherence

Table 3 shows results of bivariate tests for variation in any unintentional non-adherence across key independent variables. The proportion of respondents reporting any unintentional non-adherence was the highest among persons age 40–49 and decreased as age increased. White respondents were less likely to report any unintentional non-adherence compared to other races. Compared to respondents with education greater than college degree, respondents with a high school education or less were significantly less likely to report any unintentional non-adherence. Retired respondents reported unintentional non-adherence at the lowest rate, while those actively looking for a job reported unintentional non-adherence at the highest rate. Compared to respondents with income greater than $100,000, respondents with income less than $25,000 were significantly more likely to report any unintentional non-adherence. Reports of unintentional non-adherence decreased as self-rated health increased. Respondents with an index medication for depression had the lowest reports of unintentional non-adherence (63%) while those taking osteoporosis medications had the highest (77%). Compared to the fourth (best) quartile, persons in the first three quartiles for perceived need, concerns, and affordability scales had higher reports of any unintentional non-adherence.

**Table 3:** **Reports of*Any*Unintentional Non-Adherence across Key Variables (N = 24,017)**

	***Any*unintentional non-adherence**
	N (%)
Age^*^	
40–49	1,909 (79.0%)
50–59	6,029 (76.3%)
60–64	3,524 (71.6%)
65–69	2,629 (65.3%)
70 and up	2,741 (57.7%)
**Gender**	
Male	8,157 (70.1%)
Female	8,675 (70.0%)
Race^*^	
White	15,618 (69.7%)
Other	1,008 (76.5%)
Education^*^	
High school or less	2,803 (66.6%)
Some college	6,710 (72.0%)
College degree	2,925 (70.0%)
Greater than college degree	4,394 (70.0%)
Employment Status^*^	
Looking for a job	522 (78.9%)
Not looking for a job	1,308 (69.3%)
Retired	5,686 (62.8%)
Disabled	413 (74.5%)
Employed	8,903 (75.1%)
Income^*^	
< 25K	2,308 (73.4%)
≥ 25K < 50K	4,125 (70.4%)
≥50K < 75K	3,471 (71.1%)
≥75K < 100K	2,252 (69.2%)
≥100K	2,948 (69.9%)
Self-Rated Health^*^	
Poor/fair	5,913 (74.5%)
Good	7,104 (70.8%)
Very Good/Excellent	3,815 (63.0%)
Disease^*^	
Asthma	1,338 (75.5%)
Diabetes	2,336 (75.4%)
Hyperlipidemia	3,209 (69.2%)
Osteoporosis	2,365 (77.0%)
Depression	783 (63.4%)
Hypertension	6,801 (66.7%)
Perceived Need for Medication^*^	
Quartile 1 (Worst)	3,159 (75.5%)
Quartile 2	4,960 (74.7%)
Quartile 3	4,322 (70.7%)
Quartile 4 (Best)	4,391 (62.0%)
Perceived Medication Affordability^*^	
Quartile 1 (Worst)	4,297 (82.2%)
Quartile 2	3,774 (72.9%)
Quartile 3	5,097 (68.3%)
Quartile 4 (Best)	3,664 (59.6%)
Perceived Medication Concerns^*^	
Quartile 1 (Worst)	3,247 (77.6%)
Quartile 2	5,077 (73.6%)
Quartile 3	4,346 (70.7%)
Quartile 4 (Best)	4,162 (61.3%)

* Pearson Chi-square statistic for the omnibus test is significant at p = 0.01.

### Multivariate predictors of unintentional non-adherence

Table 4 presents results for multivariate analyses. Common multivariate predictors of each of the three unintentional non-adherence behaviors included: lower perceived need for medications, lower perceived medication affordability, younger age, worse self-rated health, and diabetes or osteoporosis (relative to hypertension). Unique predictors of ‘forgot to take medications’ were more medication concerns and male gender. Unique predictors of ‘run out of medications’ were non-white race and asthma (relative to hypertension). Unique predictors of ‘being careless’ were more medication concerns, non-white race, education greater than college degree, and asthma (relative to hypertension).

**Table 4:** Logistic Regression Models for Multivariate Predictors of Unintentional Non-Adherence (N = 24,017)

	**Forget to Take Medication**		**Run Out of Medication**		**Careless at Times Taking Medication**		***Any*unintentional non-adherence**	
	OR	CI	OR	CI	OR	CI	OR	CI
**Age**								
40–49	1.71*	1.52–1.93	2.21*	1.94–2.51	1.47*	1.28–1.68	1.95*	1.71–2.23
50–59	1.57*	1.44–1.72	2.00*	1.81–2.20	1.24*	1.11–1.37	1.78*	1.62–1.95
60–64	1.45*	1.32–1.58	1.61*	1.46–1.78	1.17^†^	1.05–1.30	1.53*	1.39–1.68
65–69	1.28*	1.17–1.40	1.29*	1.16–1.43	1.14^	1.02–1.27	1.31*	1.20–1.44
**Female gender**	.89*	.84–.94	.97	.91–1.03	1.04	.97–1.11	.91^†^	.85–.97
**White race**	.90	.80–1.02	.74*	.66–.84	.85^	.75–.96	.88	.77–1.01
**Education**								
High school or less	.79*	.72–.87	.78*	.70–.85	.77*	.69–.85	.75*	.68–.82
Some college	.94	.88–1.01	.93	.86–1.00	.91^	.84–.99	.93	.86–1.00
College degree	.93	.86–1.02	.93	.85–1.01	.90^	.82–.99	.93	.85–1.02
**Employment status**								
Looking for a job	.90	.76–1.06	1.04	.88–1.23	.95	.79–1.14	.95	.78–1.16
Not looking for a job	.74*	.67–.83	.67*	.60–.75	.75*	.67–.85	.68*	.61–.77
Retired	.78*	.73–.84	.67*	.62–.72	.75*	.69–.81	.72*	.67–.78
Disabled	.82^	.68–.98	.84	.70–1.01	.86	.70–1.05	.79^	.64–.97
**Income**								
< 25 K	.99	.90–1.09	1.74*	1.58–1.94	1.07	.96–1.20	1.21*	1.09–1.35
≥ 25K < 50K	.98	.90–1.06	1.35*	1.24–1.46	1.06	.97–1.16	1.09^	1.00–1.18
≥50K < 75K	1.05	.97–1.14	1.20*	1.10–1.30	1.03	.94–1.13	1.12^	1.03–1.22
≥75K < 100K	.98	.89–1.07	1.05	.95–1.16	1.07	.97–1.19	1.00	.91–1.10
**Self-Rated Health**								
Poor/fair	1.16*	1.07–1.25	1.70*	1.57–1.85	1.60*	1.46–1.76	1.31*	1.21–1.42
Good	1.18*	1.10–1.26	1.34*	1.24–1.45	1.33*	1.22–1.45	1.25*	1.16–1.34
**Disease**								
Asthma	.92	.83–1.03	1.36*	1.21–1.52	1.18^†^	1.05–1.33	1.18^†^	1.04–1.33
Diabetes	1.37*	1.26–1.50	1.23*	1.12–1.34	1.36*	1.24–1.50	1.38*	1.25–1.52
Hyperlipidemia	1.05	.97–1.13	.96	.89–1.04	1.08	.99–1.17	1.06	.98–1.14
Osteoporosis	1.18*	1.08–1.30	1.47*	1.34–1.61	1.30*	1.17–1.43	1.31*	1.19–1.45
Depression	.97	.85–1.10	.94	.82–1.09	1.14	.98–1.33	.95	.83–1.08
**Perceived Need for Medication**								
Quartile 1	1.46*	1.33–1.60	1.41*	1.27–1.55	1.91*	1.72–2.13	1.47*	1.32–1.62
Quartile 2	1.46*	1.35–1.57	1.38*	1.26–1.50	1.55*	1.41–1.70	1.51*	1.39–1.64
Quartile 3	1.32*	1.23–1.43	1.22*	1.20–1.32	1.34*	1.22–1.47	1.33*	1.22–1.44
**Perceived Medication Affordability**								
Quartile 1	1.29*	1.18–1.41	4.65*	4.24–5.11	1.34*	1.21–1.48	2.32*	2.10–2.55
Quartile 2	1.21*	1.11–1.31	2.30*	2.10–2.52	1.23*	1.12–1.36	1.42*	1.31–1.55
Quartile 3	1.16*	1.08–1.25	1.55*	1.42–1.69	1.10^	1.01–1.21	1.23*	1.14–1.32
**Perceived Medication Concerns**								
Quartile 1	1.25*	1.13–1.38	1.06	.95–1.17	1.25*	1.11–1.39	1.27*	1.14–1.41
Quartile 2	1.20*	1.11–1.30	1.01	.93–1.11	1.18^†^	1.08–1.31	1.19*	1.09–1.30
Quartile 3	1.20*	1.11–1.30	1.00	.92–1.09	1.14^†^	1.03–1.25	1.19*	1.10–1.29

* p < 0.001; ^†^ p < 0.01; ^ p < 0.05.

Reference Categories for each of the variables in the model were as follows: age 70 and up (Age); male (Gender); non-white (Race); greater than college degree (Education); currently employed (Employment); income > =100K (Income); very good/excellent (Self-rated health); hypertension (Disease); quartile 4 (Perceived need, Perceived affordability, Perceived concerns).

Respondents with less perceived need for medications, more medication concerns, and less perceived medication affordability were significantly more likely to report *any* unintentional non-adherence. Compared to respondents in the highest quartile for perceived need for medications, those in the first three quartiles were 33% to 51% more likely to report any unintentional non-adherence. Respondents in the lowest quartile for perceived medication affordability scores were 2.32 times more likely than those in the highest quartile to report any unintentional non-adherence. Compared to respondents in the highest quartile for perceived concerns, those in the first three quartiles were 19% to 27% more likely to report any unintentional non-adherence.

Across the three medication beliefs, perceived medication need and perceived medication affordability were stronger predictors of unintentional non-adherence than perceived medication concerns. Among demographics, age was the strongest predictor of unintentional non-adherence. Respondents in the 40–49 years age category were almost two times more likely to report any unintentional non-adherence compared to those age 70 or older.

The Adherence Estimator® total score was a significant predictor of each of the three unintentional non-adherence behaviors as well as any unintentional non-adherence. Compared to persons classified as low-risk by the Adherence Estimator® those classified as medium and high risk were significantly more likely to report forgetting medications (OR = 1.32, 1.44, respectively), carelessness with medications (OR = 1.43, 1.90, respectively), running out of medications (OR = 1.81, 1.90, respectively), and any unintentional non-adherence (OR = 1.59, 1.69, respectively).

### Mediating effect of unintentional non-adherence

It was hypothesized that the effect of medication beliefs (perceived need, perceived concerns, and perceived affordability) on intentional non-adherence is mediated through unintentional non-adherence. The four conditions outlined by Barron and Kenny for a variable to be considered as a mediating variable were satisfied (Table 5). These four conditions, demonstrated by paths a, b, c, and c’ in Figure 1, were tested through four successive linear regressions - models 1, 2, 3, and 4, respectively in Table 5. The direct effect of the three medication beliefs on unintentional non-adherence was significant (model 1). Compared to persons in the highest quartile of perceived need, those in quartiles 1, 2, and 3 scored, on average, 9.2, 7.5, and 5.1 points, respectively, higher on the unintentional non-adherence scale (with a higher score indicating a higher level of unintentional non-adherence).

**Table 5:** Unintentional Non-Adherence as a Mediator on the Direct Effect of Patient Beliefs on Intentional Non-Adherence (N = 24,017)

	**Model 1 Direct effect of patient beliefs on unintentional non-adherence**	**Model 2 Direct effect of unintentional non-adherence on intentional non-adherence**	**Model 3 Total effect of patient beliefs on intentional non-adherence**	**Model 4 Direct effect of patient beliefs on intentional non-adherence controlling for unintentional non-adherence**
	Pathway a in Figure 1	Pathway b in Figure 1	Pathway c in Figure 1	Pathway c’ in Figure 1
	DV = unintentional non-adherence	DV = intentional non-adherence	DV = intentional non-adherence	DV = intentional non-adherence
	**B (95% CI)**			
**Predictor Variables**				
**Perceived Need for Prescription**				
Quartile 1	9.2* (7.8–10.7)		4.1* (3.4–4.9)	2.7* (1.9–3.4)
Quartile 2	7.5* (6.3–8.2)		1.9* (1.3–2.6)	0.8* (0.2–1.4)
Quartile 3	5.1* (3.9–6.2)		0.6 (−0.1–1.2)	−0.2 (−0.8–0.4)
**Prescription Affordability**				
Quartile 1	18.5* (17.2–19.8)		11.9* (11.2–12.6)	9.0* (8.4–9.7)
Quartile 2	10.0* (8.7–11.3)		5.3* (4.6–5.9)	3.7* (3.1–4.4)
Quartile 3	5.1* (3.9–6.2)		1.5* (0.9–2.1)	0.7* (0.1–1.3)
**Prescription Concerns**				
Quartile 1	4.5* (3.0–6.0)		7.7* (6.9–8.5)	7.0* (6.3–7.8)
Quartile 2	3.3* (2.1–4.6)		2.5* (1.8–3.1)	2.0* (1.3–2.6)
Quartile 3	2.8* (1.6–4.0)		1.1* (0.4–1.7)	0.6* (0.1–1.2)
**Unintentional Non-Adherence**		0.2* (0.2–0.2)		0.2* (0.1–0.2)

* p < 0.05; DV = Dependent variable for the specific model being tested.

The direct effect of unintentional non-adherence on intentional non-adherence was significant (model 2), as was the total effect of medication beliefs on intentional non-adherence (model 3). Respondents with less perceived need for medications, more medication concerns, and lower perceived medication affordability had significantly higher scores on the intentional non-adherence scale (higher scores indicating greater intentional non-adherence). Consistent with the Barron and Kenny approach [40], the coefficients in model 3 were reduced after controlling for unintentional non-adherence (model 4).

The statistical significance of the mediational relationship was confirmed using bootstrapping. The bias-corrected 95% confidence intervals for the indirect effect of the three medication beliefs (perceived need, perceived concerns, and perceived affordability) were found to exclude zero. This confirmed the hypothesis that the effect of medication beliefs on intentional non-adherence is mediated through unintentional non-adherence.

## Discussion

In a sample of 24,017 adults with chronic disease who self-identified as being medication persisters, 70% reported at least one instance of unintentional non-adherence, and 34% reported at least one instance of intentional non-adherence in the past six months. Forgetfulness was the most common unintentional behavior while skipping doses to make the medication last longer was the most common intentional behavior. Our observed prevalence of unintentional non-adherence is likely higher than that reported in other studies [7,14-16,22,23,29,53-56] because of differences in the recall period as well as the wording of the question (our study asked whether the respondent “ever” demonstrated a certain behavior in the past six months, whereas several other studies asked whether the respondent “sometimes” demonstrated a certain behavior (which might be interpreted as a regular behavioral pattern).

Intentional non-adherence has been described as an active process whereby patients choose to deviate from the prescribed regimen [7]. On the other hand, unintentional non-adherence has been described as a passive process, as inadvertent or accidental medication-taking [57,58]. If unintentional non-adherence is truly random or inadvertent, then one would expect it to be weakly related to demographic characteristics and medication beliefs.

We found a linear and monotonic association between age and unintentional non-adherence: unintentional non-adherence decreased as age increased, which may possibly be attributed to multiple competing demands of professional and family life among those in their 40’s and 50’s. There is conflicting evidence from past research about the influence of age on unintentional non-adherence with some studies reporting no effect [15,20,21,28-30] and others an inverse effect similar to that reported herein [6,10]. Unlike previous studies, our large sample size allowed us to explore this relationship across several age categories. Consistent with some past research [7], individuals with lower income were more likely to report running out of medications, perhaps reflecting occasional cost-related medication underuse. Worse self-rated health was also a consistent predictor of unintentional non-adherence, which may possibly be related to a greater burden of overall medication taking across multiple comorbidities. Compared to those with hypertension, having osteoporosis or diabetes was associated with a higher likelihood of each of the three unintentional behaviors. A recent systematic review also reported that primary non-adherence, or medication non-fulfillment, was higher in patients with osteoporosis compared to hypertension [11]. Women may perceive osteoporosis to be a less serious condition compared to other diseases [59] and often do not perceive themselves to be at risk for osteoporosis [59,60], both of which may make them vulnerable to unintentional non-adherence. Diabetes is frequently accompanied by several other comorbid chronic conditions, thus increasing the pill burden of such patients and possibly making them more vulnerable to unintentional non-adherence.

Patient’s medication beliefs, especially perceived need for medication and perceived medication affordability, were strong predictors of unintentional non-adherence. Patients in the lowest quartile of perceived need for medications were 1.9 times more likely to report being careless at times with medications. Patients in the lowest quartile of perceived medication affordability were 4.65 times more likely to report running out of medications at times. Other research has also reported medication beliefs to be associated with unintentional non-adherence [15,30]. Overall, we found no evidence of unintentional non-adherence being random or accidental [21].

This study has several strengths compared to previous empirical work on unintentional non-adherence. A large, internet-based panel of adults with chronic disease was accessed. The obtained sample represents persons aged 40 and older with one of six chronic diseases from all 50 U.S. states. Our sample is by far the largest in which predictors of unintentional non-adherence has been assessed. The multi-item scales assessing perceived need for medications, perceived medication concerns, and perceived medication affordability have been previously tested and found to be reliable and valid [26]. Unlike most previous studies, the analyses were conducted in multiple chronic diseases including both physical and psychiatric disorders. Finally, the sophisticated bootstrapping approach was used to test the relationship between intentional and unintentional non-adherence.

Despite these strengths, several limitations must be taken in to account when interpreting the results. This study was conducted over the Internet with 29.5% survey contact rate. The CIP recruits panel members through multiple avenues in an attempt to provide a representative sample of the adult, chronically-ill population and to reach under-represented and hard-to-reach populations of interest. However, some biases were observed in the sample. Specifically, compared to the U.S. adult population, the obtained sample was under-represented by adults with income less than $25,000 annually [61], under-represented by adults with a high school education or less [62], and over-represented by Caucasians [63]. As a result, generalizability to the broader population should be made with caution. Because the obtained sample is under-represented by persons of lower income relative to the U.S. adult population, it is likely that the observed effects of income and perceived medication affordability represent lower-bound estimates. All respondents were self-identified persisters to prescription medications, and we did not confirm this based on pharmacy-claims data or other adherence metrics (such as pill counts). The unintentional non-adherence items were dichotomous (yes/no); we did not inquire about the frequency of each unintentional medication-taking behavior. Finally, this study had a cross-sectional design. Results from this study, especially the relationship between intentional and unintentional non-adherence, should be confirmed using longitudinal data.

Numerous measures assessing unintentional non-adherence exist. For example, the four-item Morisky Scale [35] contains two items on unintentional non-adherence and the eight-item Morisky Scale [64] contains three unintentional items. However, an interpretative problem with most measures of unintentional non-adherence is that they do not reveal the underlying *reasons* for forgetfulness or carelessness. The analyses reported herein suggest that unintentional non-adherence is influenced by the same drivers as intentional non-adherence—patients’ beliefs about the medications. While unintentional non-adherence may appear to be a rather benign behavior, its importance lies in its potential prognostic significance. Qualitative research has depicted how patients test or experiment with their medications [9,65,66]. It is plausible that acts of unintentional non-adherence may be prescient of future intentional non-adherence. Our meditational tests support this hypothesis. With occasional episodes of unintentional non-adherence, patients may be testing a medication’s effectiveness or gauging symptom status without the medication. Others have suggested that reports of forgetfulness in taking medications may be a proxy for reduced motivation [67], having doubts about the prescribed therapy [68], or having low perceived need for the medication [29,69]. Our analyses support these interpretation as all three medication beliefs scales were significantly associated with reports of forgetfulness, carelessness, and any unintentional non-adherence.

Non-persistence most commonly occurs in the first six months of therapy [70-77]. It is during this initial period that both unintentional and intentional non-adherence should be assessed. Because physicians poorly predict patients’ adherence [78,79], adherence screeners, like the Adherence Estimator® [26], might prove useful in identifying nascent, suboptimal medication beliefs that may lead to forgetfulness, carelessness, and, perhaps eventually, non-persistence all together.

## Conclusions

For our study sample, unintentional non-adherence does not appear to be random and is predicted by medication beliefs, chronic disease, and sociodemographics. Our data suggests that the importance of unintentional non-adherence may lie in its potential prognostic significance for future intentional non-adherence. Health care providers may consider routinely inquiring about unintentional non-adherence in order to proactively address patients’ suboptimal medication beliefs before they choose to discontinue therapy all together.

## Competing interests

Drs. Gadkari and McHorney are full-time employees of and own stock in Merck & Co., Inc.

## Authors’ contributions

Both AG and CM contributed to the conception of the manuscript idea. CM led the instrument development and data collection process. AG led the statistical analysis and interpretation of data for the manuscript. Both AG and CM contributed to the original draft of the manuscript as well as to the critical revision of the manuscript for important intellectual content. Both AG and CM have given final approval for the current version of the manuscript to be published.

## Pre-publication history

The pre-publication history for this paper can be accessed here:

http://www.biomedcentral.com/1472-6963/12/98/prepub
